# Case Report: A case of femoral metastatic cancer misdiagnosed as isolated femoral lesser trochanter avulsion fracture

**DOI:** 10.3389/fonc.2025.1565771

**Published:** 2025-06-17

**Authors:** Meng-qi Pang, Feng-ze Wu, Jia-yan Chen, Rang-teng Zhu, Gang Jin, Wei-jie Gong, Han-tao Jiang

**Affiliations:** ^1^ Department of Clinical Laboratory, Taizhou Hospital of Zhejiang Province Affiliated to Wenzhou Medical, Taizhou, Zhejiang, China; ^2^ Department of Orthopedics, Taizhou Hospital of Zhejiang Province Affiliated to Wenzhou Medical, Taizhou, Zhejiang, China

**Keywords:** metastatic, isolated femoral lesser trochanter fracture, computed tomography, magnetic resonance imaging, case report

## Abstract

**Objective:**

To highlight the diagnostic challenges and clinical implications of metastatic disease presenting as an atypical fracture in a patient with a history of lung cancer, emphasizing the importance of maintaining a high index of suspicion for metastatic disease and the need for comprehensive diagnostic approaches.

**Methods:**

We present a case of a 79-year-old male with a history of poorly differentiated squamous cell carcinoma of the left lung who presented with left hip pain after minor trauma. Initial X-ray and CT imaging suggested an avulsion fracture of the femoral lesser trochanter(LT).The patient was managed conservatively with bed rest. However, persistent pain led to further evaluation with MRI, revealing an underlying pathological fracture due to metastatic cancer.

**Results:**

Initial radiographic and CT findings showed a localized bone defect and surrounding soft tissue swelling, consistent with an avulsion fracture of the LT. However, MRI and contrast-enhanced MRI revealed irregularities in the femoral LT with abnormal bone marrow signals and a prominent soft tissue mass, leading to the diagnosis of a pathological fracture secondary to metastatic cancer. This case underscores the limitations of initial imaging modalities in detecting subtle bone marrow changes and the importance of MRI in identifying metastatic lesions.

**Conclusion:**

The misdiagnosis of a pathological fracture as an avulsion fracture can have significant clinical implications, including increased morbidity and delayed treatment of metastatic disease. This case highlights the importance of maintaining a high index of suspicion for metastatic disease, especially in patients with a history of malignancy, and the need for comprehensive diagnostic approaches, including MRI to avoid misdiagnosis. Early recognition and appropriate management of pathological fractures are crucial for improving patient outcomes and quality of life.

## 1 Introduction

An isolated avulsion fracture of the femoral lesser trochanter (LT) is a relatively uncommon yet clinically significant orthopedic injury (1). The lesser trochanter is a bony prominence located on the proximal posterior aspect of the femur and serves as a major attachment site for the iliopsoas muscle (2). In young individuals, LT fractures typically result from high-impact trauma, such as that experienced in contact sports, where a direct blow to the hip may cause avulsion of the LT (3). However, in the elderly, such fractures often serve as a “red flag” for underlying malignancy, particularly metastatic cancer (4). Given the potential for serious systemic disease, it is critical to conduct comprehensive evaluations—such as imaging studies and biopsies—when elderly patients present with isolated LT fractures.

While rare in adults, isolated avulsion fractures of the LT are often indicative of an underlying pathological condition, most commonly malignancies such as metastatic lesions or primary bone tumors (1). The majority of these cases occur in older adults, with metastatic fractures being the most prevalent (5). Diagnosis typically involves imaging modalities including X-rays and MRI, and in cases without a known cancer history, a biopsy may be required to confirm the presence of malignancy (6). Treatment strategies vary based on the underlying etiology: surgical wide resection and tumor prosthesis reconstruction are generally recommended for primary malignancies, while palliative interventions such as prophylactic intramedullary nailing or radiation therapy may be considered for metastatic disease (7). In hypervascular tumors, preoperative embolization can help reduce intraoperative blood loss (8). Patient prognosis is largely dictated by the type and stage of the primary malignancy, although advancements in chemotherapy and targeted therapies have significantly improved survival rates (9). A systematic and stepwise approach to diagnosis and treatment is crucial to avoid misdiagnosis and ensure optimal clinical management.

Despite its clinical importance, isolated LT avulsion fractures are often under-recognized due to their low incidence. This lack of awareness among some emergency physicians may lead to misdiagnosis or delayed diagnosis (10), which in turn can result in inappropriate treatment, failure to initiate timely intervention, and ultimately poorer patient outcomes.

This case report highlights the importance of maintaining a high index of suspicion for pathological causes of LT fractures. We present a patient with a previously undocumented pulmonary tumor who experienced hip pain following physical exertion. Initial imaging including X-ray and CT scan suggested an isolated LT fracture, prompting conservative treatment with bed rest and subsequent MRI evaluation. However, the MRI ultimately revealed the presence of a metastatic carcinoma in the region of the lesser trochanter, underscoring the need for thorough diagnostic workup to distinguish between traumatic and pathological fractures—particularly in atypical presentations.

## 2 Presentation of the case

A 79-year-old male with a history of smoking was admitted to our hospital for evaluation of a left pulmonary mass detected on routine chest radiography. On February 20, 2024, a percutaneous biopsy of the pulmonary mass was performed, and histopathological examination confirmed the diagnosis of poorly differentiated squamous cell carcinoma of the left lung. A whole-body bone scan conducted on February 21, 2024, showed no evidence of bone metastasis. The patient was initiated on first-line chemotherapy on March 8, 2024, consisting of carboplatin 300 mg on day 1, paclitaxel 270 mg on day 1, and tislelizumab 200 mg on day 1, administered in a 21-day cycle. The patient tolerated the chemotherapy regimen well without significant adverse events.

**Figure 1:**
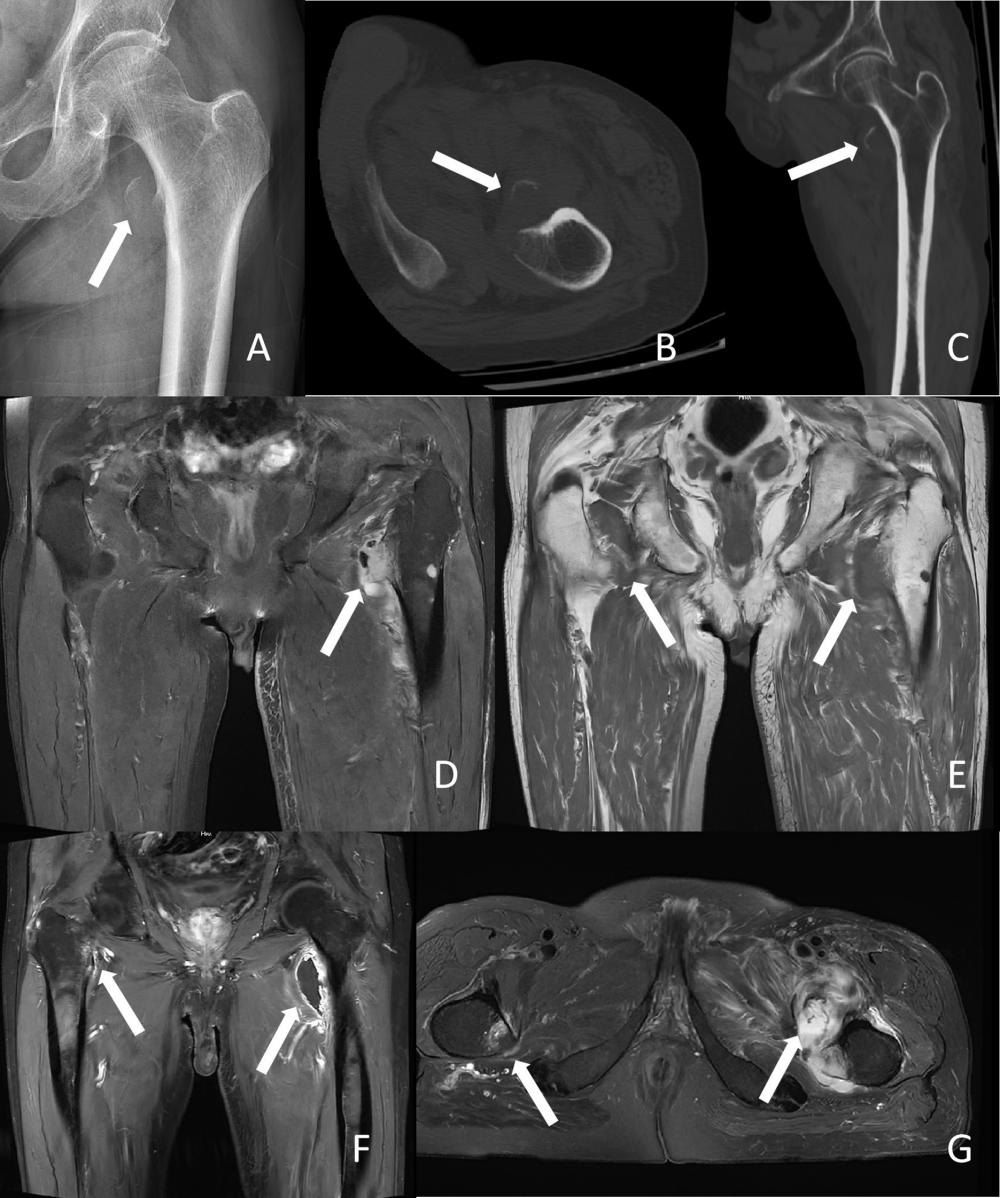
Radiological images of the patient. **(A)** X-ray findings. The white arrow indicates the fracture site of the left lesser trochanter. **(B, C)** Computed tomography scan results. The white arrow indicates the fracture site of the left lesser trochanter. **(D)** Magnetic resonance imaging (MRI) T2-weighted imaging results. The white arrow indicates destruction of the left lesser trochanter, suggestive of a pathological fracture. **(E)** MRI T1-weighted imaging results. The white arrow indicates both lesser trochanters, suggestive of metastatic tumors in both lesser trochanters. **(F, G)** Contrast-enhanced MRI results. The white arrow indicates both lesser trochanters, suggestive of metastatic tumors in both lesser trochanters.

On November 3, 2024, the patient presented to our emergency department with left hip pain and limited mobility after riding a bicycle. Initial X-ray examination revealed a nodular bone fragment adjacent to the left femoral LT (**Figure 1A**), suggestive of an avulsion fracture of the left femoral LT. A subsequent CT scan showed a localized bone defect in the left femoral LT with a patchy hyperdense shadow anteriorly and surrounding soft tissue swelling (**Figures 1B, C**), consistent with the diagnosis of an avulsion fracture. Given the patient’s advanced age and recent history of hip loading, coupled with the absence of bone metastasis on a whole-body bone scan performed six months prior, the patient was diagnosed with an avulsion fracture of the lesser trochanter and managed conservatively with bed rest.

After two weeks of bed rest, the patient’s pain persisted and progressively worsened, prompting a visit to our orthopedic outpatient clinic. Considering the patient’s history of pulmonary malignancy, further evaluation was warranted to rule out pathological fracture. A MRI scan of the left hip was performed, which revealed irregularity of the left femoral LT with abnormal bone marrow signal, showing patchy high signal intensity on proton density fat-saturated (PDFS) sequences and surrounding soft tissue swelling (**Figures 1D, E**). The imaging diagnosis was avulsion fracture of the left femoral lesser trochanter with associated soft tissue injury. To further clarify the diagnosis, a contrast-enhanced MRI of both hip joints was performed. The enhanced MRI demonstrated nodular abnormal signals in the lesser trochanters of both femurs and the lower part of the left femoral trochanter, with low signal intensity on T1-weighted imaging (T1WI) and high signal intensity on T2-weighted imaging with fat suppression (T2WI+FS). The lesion in the left femoral lesser trochanter was more prominent, measuring approximately 22 mm, with an associated soft tissue mass. The final imaging diagnosis was abnormal bone marrow signals in the lesser trochanters of both femurs and the lower part of the left femoral trochanter, with a prominent soft tissue mass in the left femoral lesser trochanter (**Figures 1F, G**). The patient was ultimately diagnosed with a pathological fracture of the proximal left femur secondary to metastatic cancer.

Since the patient had undergone whole abdominal and chest CT scans within the past week, which showed no intra-abdominal tumors, and the bone scan also revealed no other primary bone tumors, we concluded that the primary site of the metastatic cancer in the lesser trochanter of the femur was a poorly differentiated squamous cell carcinoma of the lung. Given the patient’s advanced age and poor surgical tolerance, palliative radiotherapy was administered for the metastatic lesions in both femurs. The poorly differentiated squamous cell carcinoma of the lung continued to be treated with chemotherapy combined with immunotherapy using “carboplatin + paclitaxel + tislelizumab.” Unfortunately, after receiving palliative radiotherapy, the patient developed a pulmonary infection, which was treated with cefoperazone-sulbactam and moxifloxacin. However, the treatment was not effective, and the patient ultimately succumbed to severe pulmonary infection.

## 3 Discussion

The case presented here highlights the diagnostic challenges and clinical implications of metastatic disease presenting as an atypical fracture in a patient with a history of lung cancer. Despite initial evaluations suggesting an avulsion fracture of the left femoral LT, further imaging and clinical reassessment revealed an underlying pathological fracture due to metastatic cancer. This case underscores the importance of maintaining a high index of suspicion for metastatic disease, especially in patients with a history of malignancy, and the need for comprehensive diagnostic approaches to avoid misdiagnosis.

### 3.1 Clinical presentation and initial diagnosis

The patient initially presented with left hip pain and limited mobility following minor trauma sustained while bicycling. These clinical manifestations were suggestive of an avulsion fracture of the LT. Avulsion fractures of the LT are relatively uncommon, typically occurring in younger individuals due to direct trauma or high-impact activities (3). However, in this case, the patient’s advanced age and the absence of bone metastasis on a whole-body bone scan performed six months prior further supported the initial diagnosis of an avulsion fracture. Radiographic and CT findings were consistent with this diagnosis, revealing a localized bone defect and surrounding soft tissue swelling. These imaging findings aligned with the typical presentation of avulsion fractures, which often show a fragment of bone being pulled away from its main body by soft tissue attachments (10).

However, as pointed out by Petrova et al. (11) in their study of a rare case of sural schwannoma, the clinical manifestations of certain diseases may be masked by their nonspecific symptoms - in that study, the schwannoma’s slow growth and insidious symptoms led to initial diagnostic difficulties; similarly, in the present case, although the imaging findings were consistent with an avulsion fracture, the patient’s history of malignant tumor indicated the need for a more comprehensive evaluation. Subsequent disease progression and further imaging examinations revealed a metastatic lesion in the proximal femur, ultimately leading to a diagnosis of pathological fracture caused by metastatic cancer (12, 13). This process highlights the critical importance of maintaining a high index of suspicion in patients with malignant tumors, even when initial imaging suggests benign lesions.

This case underscores the diagnostic challenges posed by atypical presentations, particularly in elderly patients or those with malignancy histories, where pathological fractures may mimic traumatic injuries. While avulsion fractures are rare in older adults, their presentation can overlap with metastatic lesions, necessitating a high index of suspicion (1). The literature highlights that metastatic fractures often present with similar acute symptoms but are associated with a history of cancer or systemic symptoms (14). In this case, the absence of prior malignancy documentation delayed the correct diagnosis, emphasizing the need for comprehensive clinical evaluation, including metastatic workup in elderly patients with unexplained fractures.

### 3.2 Diagnostic pitfalls and re-evaluation

Despite the initial diagnosis, the persistence and worsening of pain after two weeks of conservative management raised concerns about the possibility of an underlying pathological fracture. The patient’s medical history of poorly differentiated squamous cell carcinoma of the left lung—a malignancy recognized for its aggressive behavior and high metastatic potential-further supported the need for more comprehensive evaluation (15, 16).

Initial imaging, including plain radiography and CT, may often fail to detect early bone marrow involvement, especially in cases of metastatic disease (17, 18). In this case, MRI emerged as a critical diagnostic tool due to its superior sensitivity in identifying subtle marrow abnormalities. The MRI findings revealed irregular signals within the left femoral lesser trochanter (LT), accompanied by surrounding soft tissue swelling—an appearance inconsistent with a simple avulsion fracture and suggestive of an underlying pathological process (19).

Contrast-enhanced MRI subsequently demonstrated nodular lesions in both femoral LTs along with a prominent soft tissue mass in the left side, findings that are highly characteristic of metastatic involvement (12). These imaging characteristics are consistent with previous case reports describing metastatic lesions to the femoral LT, which often present as ill-defined areas of signal alteration on T1-weighted images and enhancement on post-contrast sequences (20). Ultimately, these findings led to the diagnosis of a pathological fracture secondary to metastatic carcinoma.

This case underscores the diagnostic limitations of conventional radiographic and CT imaging in detecting early osseous metastases and emphasizes the pivotal role of MRI in identifying subtle marrow changes and confirming metastatic disease, particularly in patients with known primary malignancies and atypical musculoskeletal symptoms.

### 3.3 Clinical implications and management

The misdiagnosis of a pathological fracture as an avulsion fracture carries substantial clinical consequences. Delayed identification and treatment of underlying metastatic disease can exacerbate morbidity, including progressive pain, risk of additional fractures, and diminished quality of life (21). In this case, the initial conservative management—bed rest—was appropriate for an avulsion fracture but inadequate for addressing the metastatic etiology. Timely reassessment and accurate diagnosis facilitated a pivotal shift in therapeutic strategy toward managing the metastatic lesions.

The management of pathological fractures secondary to metastatic disease typically requires a multidisciplinary approach, integrating oncology, orthopedics, and radiology (22). Treatment modalities may encompass surgical stabilization, radiation therapy, and systemic anticancer therapies (22, 23). In this instance, the patient’s treatment regimen necessitated modification to target the newly identified femoral LT metastases.

This case underscores the diagnostic intricacies of metastatic disease manifesting as an atypical fracture. While the initial diagnosis of an LT avulsion fracture was clinically plausible based on presentation and imaging, the persistence of symptoms—coupled with the patient’s malignancy history—warranted further investigation. Ultimately, this revealed a pathological fracture secondary to metastasis. Our findings align with prior reports highlighting the diagnostic challenges posed by metastatic fractures, which may mimic benign lesions due to overlapping imaging characteristics (1, 24). For example, Potter et al. (24) described a case where a pathological fracture was initially misdiagnosed as a stress fracture in a patient with occult breast cancer, emphasizing the need for vigilance in oncology patients. Similarly, Cho et al. (1) reported a series of metastatic fractures initially overlooked due to atypical radiographic features, reinforcing the importance of MRI in detecting subtle bone marrow changes.

This case reinforces the critical importance of maintaining a high index of suspicion for metastatic disease in patients with malignancy histories, even when initial imaging suggests a benign etiology. Comprehensive diagnostic strategies—including advanced imaging such as MRI—are essential to avoid misdiagnosis. Early recognition and appropriate management of pathological fractures are paramount for optimizing patient outcomes and quality of life.

## Data Availability

The original contributions presented in the study are included in the article/supplementary material. Further inquiries can be directed to the corresponding author.
